# Supplementary material for: A non-canonical visual cortical-entorhinal pathway contributes to spatial navigation
Source: Nat Commun. 2024 May 15;15:4122. doi: 10.1038/s41467-024-48483-y (PMC11096324; doi:10.1038/s41467-024-48483-y)
Supplement: Supplementary file 1 — Supplementary Information [file 41467_2024_48483_MOESM1_ESM.pdf]

SUPPLEMENTARY INFORMATION

A non-canonical visual cortical-entorhinal pathway contributes to spatial navigation

Supplementary Figures: 12

Qiming Shao[1], Ligu Chen[1], Xiaowan Li[1], Miao Li[1], Hui Cui[1], Xiaoyue Li[1], Xinran Zhao[1], Yuying Shi[1], Qiang Sun[1], Kaiyue Yan[1], Guangfu Wang[1]

[1]HIT Center for Life Sciences, School of Life Science and Technology, Harbin Institute of Technology, Harbin 150001, China.

Address for correspondence:
Guangfu Wang
HIT Center for Life Sciences
School of Life Science and Technology
Harbin Institute of Technology
92 West Dazhi Street
Harbin 150001, China

Email: wangguangfu@hit.edu.cn

Supplementary Figure 1

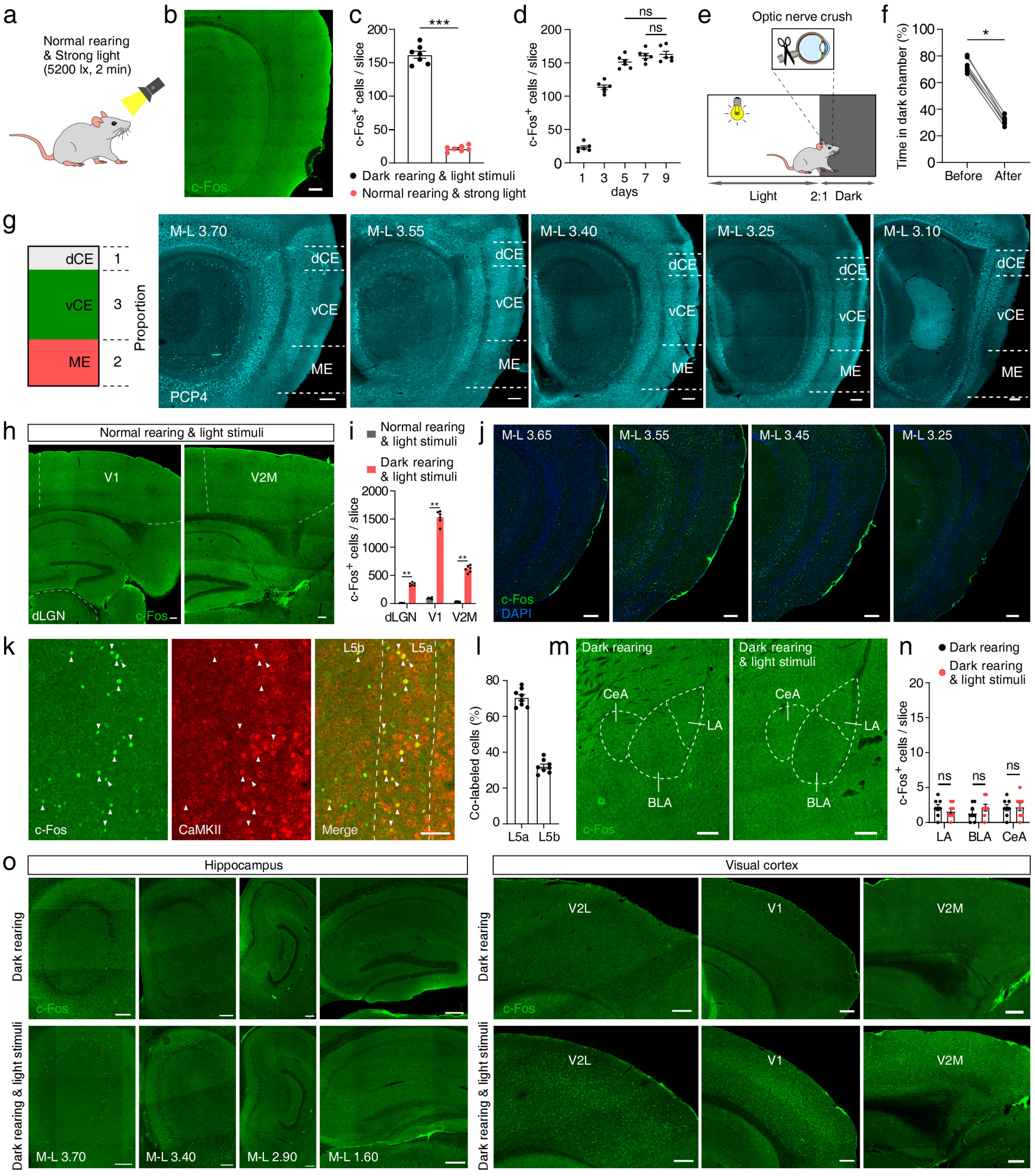

Supplementary Figure 1, related to Figure 1. Major brain areas that respond to brief visual stimuli.
a, Schematic illustrating normally reared mice given a stronger light stimulus.
b, Confocal image showing the corresponding c-Fos expression.
c, Comparison of c-Fos$^+$ cell numbers in the MEC for the mouse groups of dark rearing with light stimuli and normal rearing with stronger light stimuli (n = 7 mice/group; ***P = 0.0006).
d, The change of c-Fos expression in the MEC with days of dark rearing before light stimuli (n = 6 slices from 3 mice/group; ns $P \geq 0.05$).
e, Schematic illustrating optic nerve crushing and light/dark preference test.
f, Proportion of time spent in the dark chamber before and after optic nerve crushing (n = 7 mice; *P = 0.0156).
g, Sagittal sections showing CE and ME subdivisions of the MEC, with the former further divided into dorsal 1/4 (dCE) and ventral 3/4 parts (vCE).
h, Confocal image showing c-Fos expression in the visual cortex for normal reared mice with light stimuli.
i, Comparison of c-Fos$^+$ cell numbers in the MEC for the mouse groups of dark rearing and normal rearing with light stimuli (n = 6 mice/group; **P = 0.0022).
j, Sagittal sections showing c-Fos expression in MEC L5a along the mediolateral (ML) axis.
k, Confocal images showing c-Fos$^+$ cells co-labeled with CaMKII antibody in MEC L5.
l, Proportion of co-labeled cells in c-Fos$^+$ cells for MEC L5 (n = 8 slices from 4 mice).
m, Coronal sections showing c-Fos expression in the lateral (LA), basolateral (BLA) and central amygdala (CeA) for dark reared mice without/with light stimuli.
n, Comparison of c-Fos$^+$ cell numbers in the amygdala for dark reared mice without/with light stimuli (n = 8 slices from 4 mice/group; ns $P \geq 0.05$).
o, Confocal images showing c-Fos expression in the visual cortical areas and the hippocampus for dark reared mice without/with light stimulus.
Scale bars, 50 μm (**k**), 200 μm (others). Two-sided Wilcoxon signed-rank test (**c**, **d**, **i** and **n**) or Mann-Whitney U test (**f**). Error bars represent SEM. Source data are provided as a Source Data file.

Supplementary Figure 2

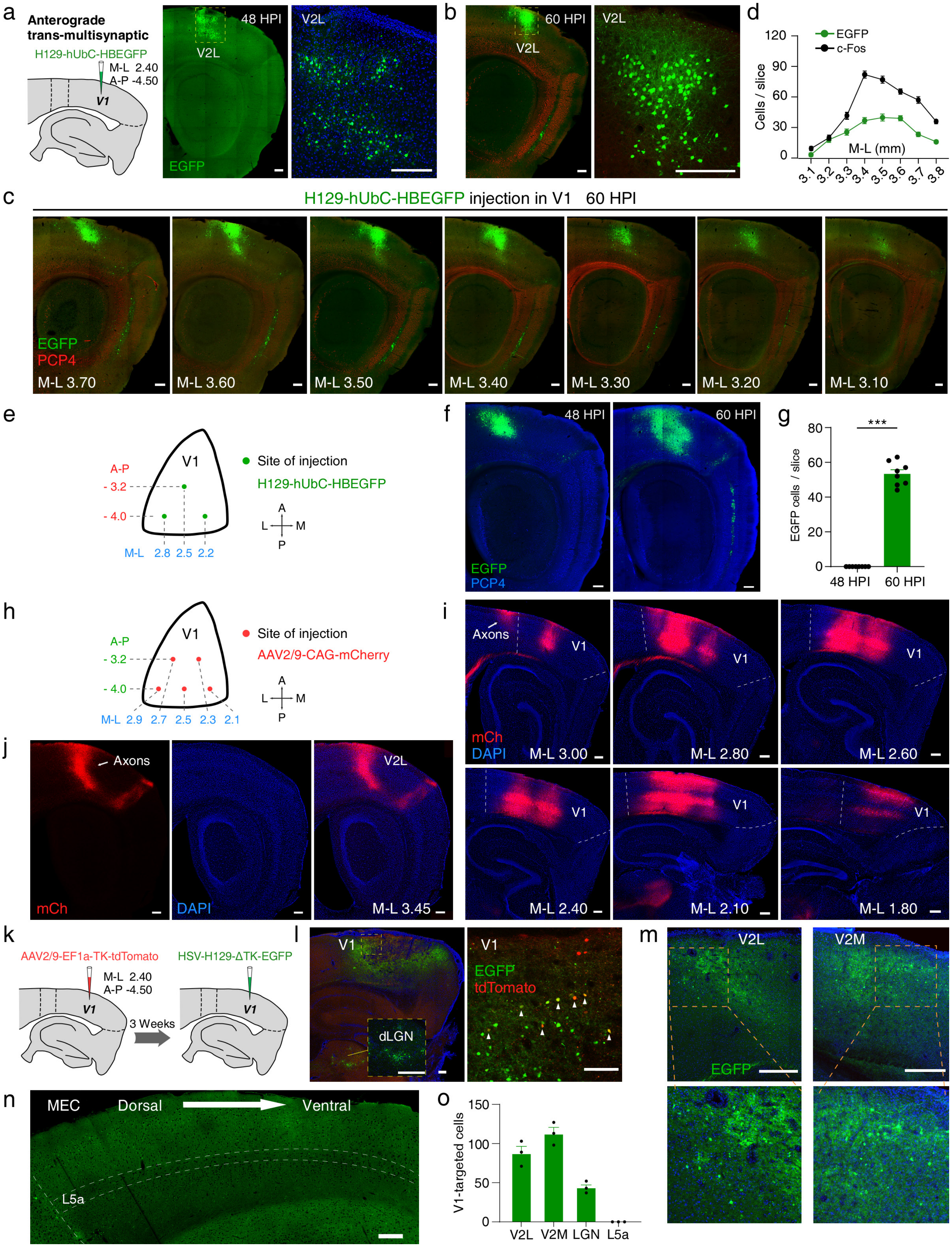

Supplementary Figure 2, related to Figure 2. V1 has indirect connectivity to MEC L5a.
a, Left: schematic illustrating anterograde trans-multisynaptic HSV tracing strategy. H129-hUbC-HBEGFP was injected in V1. Right: confocal images showing HSV1 H129 expression at 48 HPI within the V2L.
b, Confocal images showing HSV1 H129 expression at 60 HPI within the V2L.
c, Sagittal sections showing the change of trans-multisynaptic HSV1 H129 expression at 60 HPI in MEC L5a along the ML axis. Note that the virus was injected in V1.
d, Distribution of c-Fos^+ cells induced by light stimulus and HSV expression in MEC L5a along the ML direction (n = 3 mice).
e, Schematic illustrating injection of HSV1 H129 at multiple sites of V1.
f, Sagittal sections showing the HSV1 H129 expression at 48 HPI or 60 HPI within the MEC.
g, Comparison of HSV1 H129 expression within the MEC L5a at 48 HPI and 60 HPI (n = 8 slices from 4 mice/group). ***P = 0.0002, two-sided Mann-Whitney U test.
h, Schematic illustrating injection of AAV2/9-CAG-mCherry at multiple sites of V1.
i, Sagittal sections showing the AAV2/9-CAG-mCherry expression in V1 at different ML positions.
j, Sagittal sections showing that V1 projections were not present in the MEC.
k, Schematic illustrating viral injection within V1 for anterograde trans-monosynaptic HSV tracing.
l, Left, confocal images showing viral expression within the V1. Right, starter cells (yellow, arrowheads) co-expressing AAV2/9-EF1α-TK-tdTomato (red) and H129-ΔTK-EGFP (green).
m, Confocal images showing HSV expression within the V2L and V2M.
n, Confocal images showing HSV expression within the MEC.
o, Number of $EGFP^+$ cells in the V2L, V2M, LGN and MEC L5a (n = 3 slices from 3 mice/group).
Scale bars, 200 μm. Error bars represent SEM. Source data are provided as a Source Data file.

Supplementary Figure 3

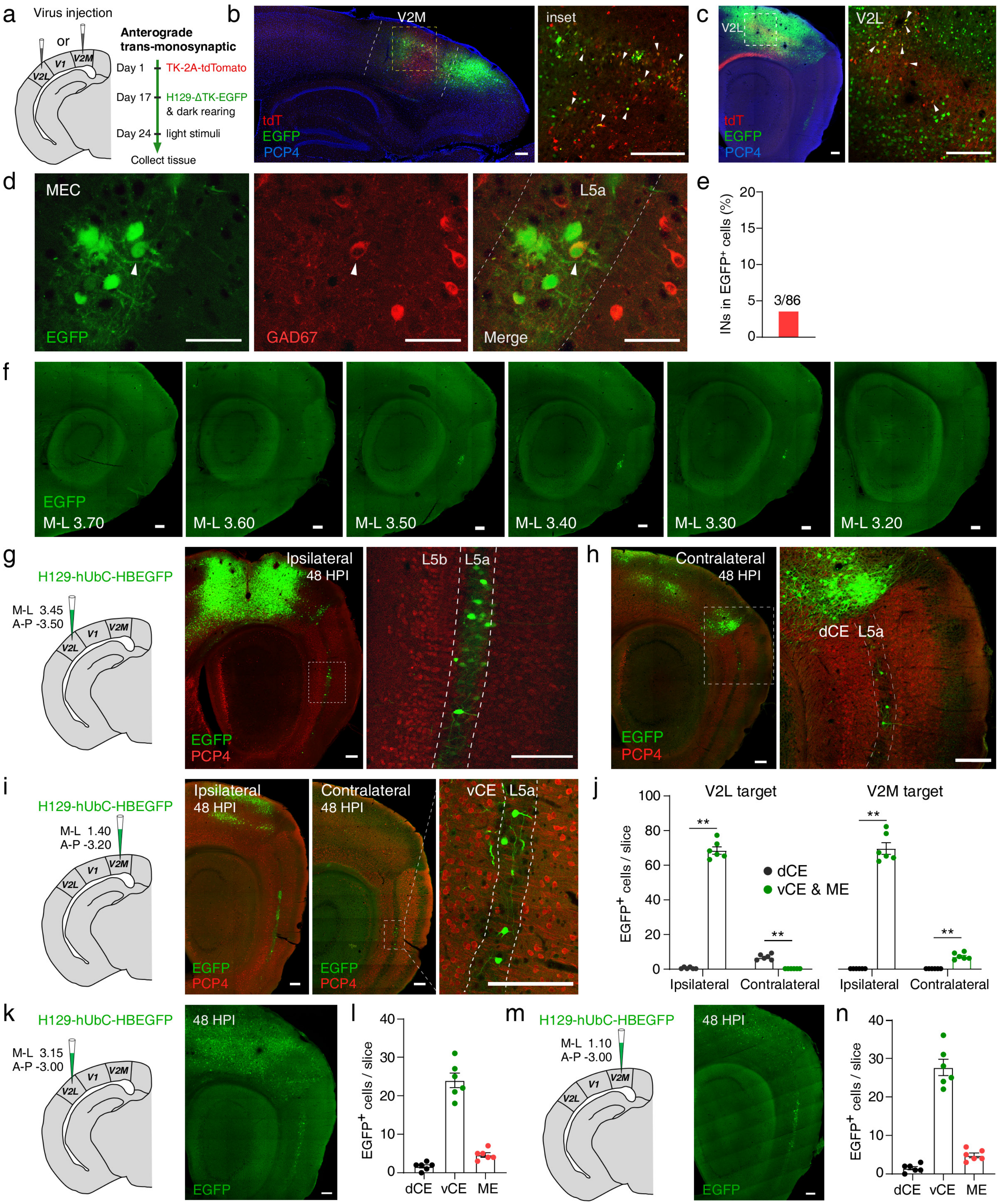

Supplementary Figure 3, related to Figure 2. V2 has monosynaptic connectivity to MEC L5a.

a, Schematic illustrating anterograde trans-monosynaptic HSV tracing and light stimulation strategy. AAV2/9-EF1α-TK-tdTomato was injected in V2M or V2L on day 1, and H129-ΔTK-EGFP on day 17. Mice received light stimulus on day 24.

b, Left: confocal image showing viral expression within the V2M. Right: starter cells (yellow, arrowheads) co-expressing AAV2/9-EF1α-TK-tdTomato (red) and H129-ΔTK-EGFP (green).

c, Left: confocal image showing viral expression within the V2L. Right: starter cells (yellow, arrowheads) co-expressing AAV2/9-EF1α-TK-tdTomato (red) and H129-ΔTK-EGFP (green).

d, Confocal images showing HSV expression in V2M-targeted interneurons (INs) of vCE L5a.

e, Proportion of INs in $EGFP^+$ cells within MEC L5a (n = 5 slices from 3 mice).

f, Sagittal sections showing the change of EGFP expression in MEC L5a along the ML axis.

g, Left: schematic illustrating viral injection within V2L for anterograde HSV tracing. Right: confocal images showing HSV expression within the ipsilateral MEC at 48 HPI.

h, Confocal images showing HSV expression within the contralateral MEC at 48 HPI.

i, Left: schematic illustrating viral injection within V2L for anterograde HSV tracing. Right: confocal images showing HSV expression within the bilateral MEC at 48 HPI.

j, Comparison of HSV expression within bilateral MEC L5a at 48 HPI (n = 6 slices from 3 mice). ***P* = 0.0022, two-sided Mann-Whitney U test. Error bars represent SEM.

k, HSV1 H129 was injected more medially and anteriorly in V2L compared to **g**.

l, Comparison of HSV expression within dCE, vCE, ME of MEC L5a at 48 HPI (n = 6 slices from 3 mice).

m, HSV1 H129 was injected more medially and anteriorly in V2M compared to **i**.

n, Comparison of HSV expression within dCE, vCE, ME of MEC L5a at 48 HPI (n = 6 slices from 3 mice).

Scale bars, 50 μm (**d**), 200 μm (others). Source data are provided as a Source Data file.

Supplementary Figure 4

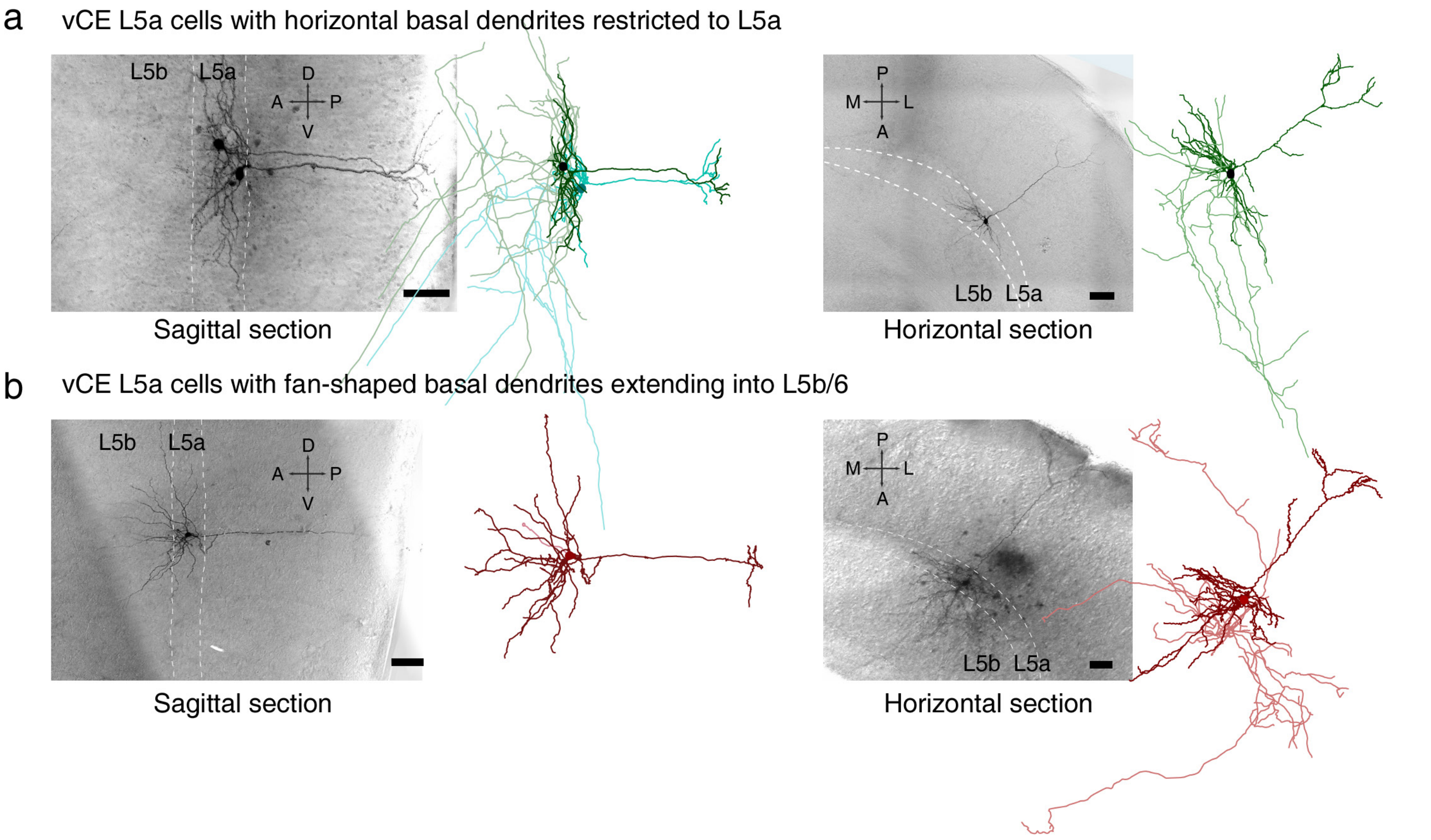

Supplementary Figure 4, related to Figure 2. Morphologies of MEC L5a neurons targeted by V2M and V2L.
a-b, Morphological reconstructions of vCE L5a PCs with horizontal (**a**) or fan-shaped (**b**) basal dendrites in sagittal (left) and horizontal (right) sections (lines in light color represent axons). Scale bars, 100 µm.

Supplementary Figure 5

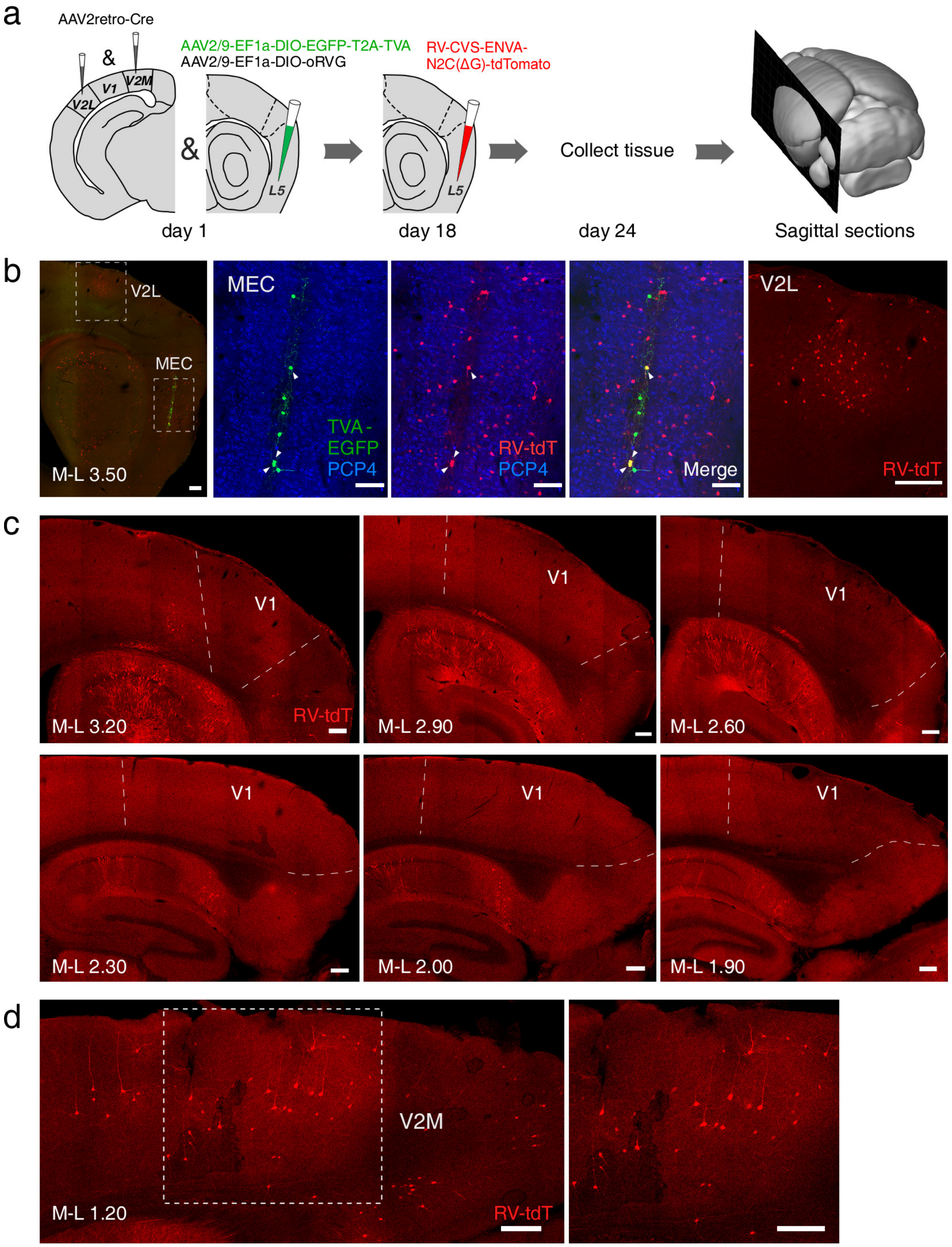

Supplementary Figure 5, related to Figure 3. V2M and V2L contribute to V2→MEC L5a pathway.
a, Schematic illustrating strategy of retrograde trans-monosynaptic RV tracing from MEC.
b, Sagittal sections showing RV expression in MEC and V2L. Note starter cells (yellow, arrowheads).
c, Sagittal sections showing the RV expression in V1 at different ML positions.
d, Sagittal sections showing RV expression in V2M.
Scale bars, 200 μm.

Supplementary Figure 6

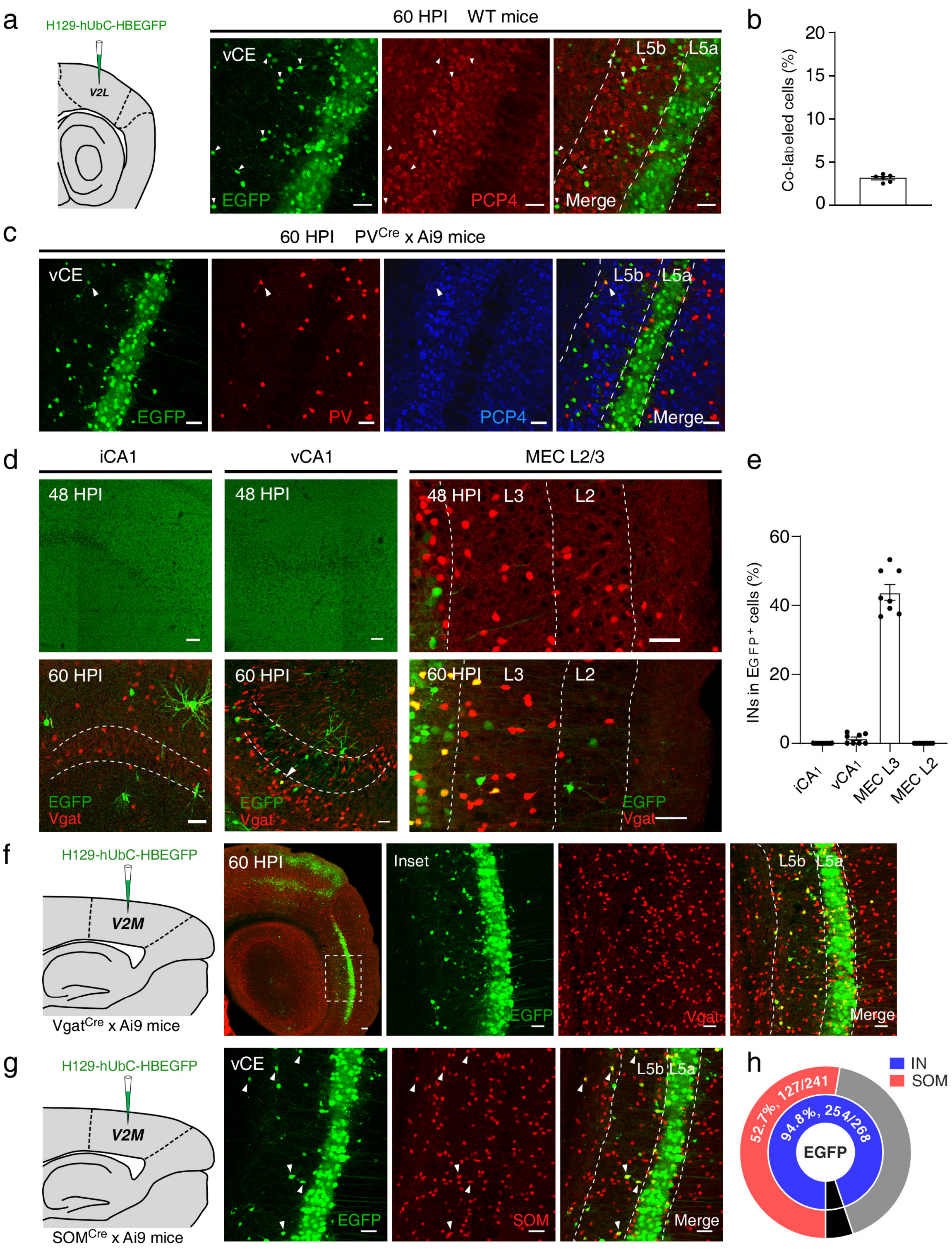

Supplementary Figure 6, related to Figure 5. Downstream targets of the V2→MEC L5a pathway in the MEC and the hippocampus.

a, Left: schematic illustrating anterograde trans-multisynaptic HSV tracing strategy. Right: confocal images showing vCE L5 and L6 neurons expressing H129-EGFP (green) and PCP4 antibody (red) at 60 HPI in WT mice. Arrowheads indicate partial EGFP-positive/PCP4-negative cells.

b, Proportion of PCP4-positive cells in EGFP-positive cells (*n* = 6 slices from 3 mice).

c, Confocal images showing vCE L5a neurons expressing H129-EGFP (green) at 60 HPI in PV^{Cre} x Ai9 mice. The arrowhead indicates co-expression of EGFP and tdTomato.

d, Confocal images showing H129-EGFP (green) expression within the ld/vCA1 and MEC L2/3 at 48 or 60 HPI.

e, Proportion of INs in EGFP-positive cells of ld/vCA1 or MEC L2/3 at 60 HPI (*n* = 8 slices from 4 mice).

f-g, Left: schematic illustrating anterograde trans-multisynaptic HSV tracing strategy. Virus injections within V2M were performed in $Vgat^{Cre}$ x Ai9 (**f**) and SOM^{Cre} x Ai9 (**g**) mice. Right: Confocal images showing H129-EGFP (green) expression within MEC L5a at 60 HPI. Arrowheads indicate co-labeled cells.

h, Proportions of Vgat-positive and SOM-positive cells in EGFP-positive cells within MEC L5b/6 (*n* = 3 mice/group).

Scale bars, 50 μm. Error bars represent SEM. Source data are provided as a Source Data file.

Supplementary Figure 7

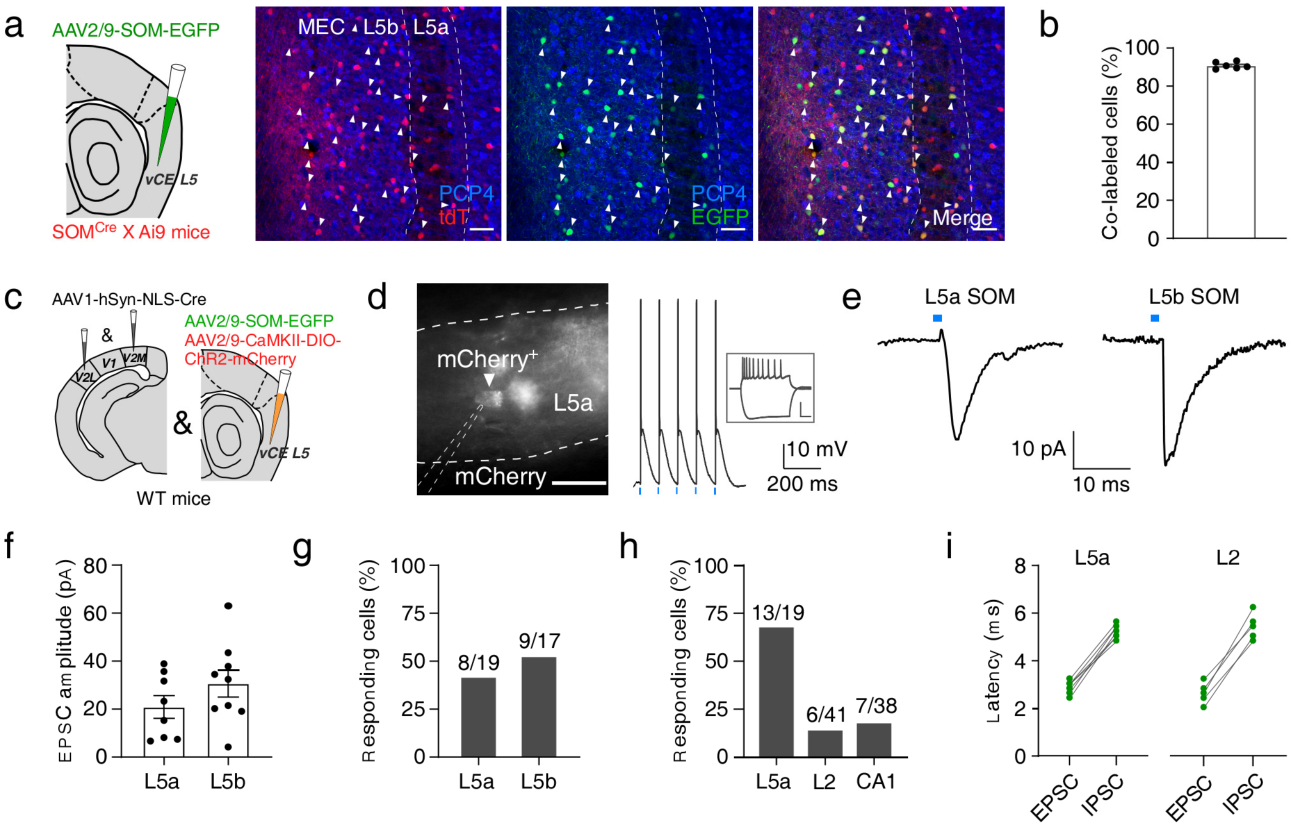

Supplementary Figure 7, related to Figure 5. Downstream targets of the V2→MEC L5a pathway in the MEC and the hippocampus.

a, Left: schematic illustrating AAV2/9-SOM-EGFP injection in vCE L5 of SOM^{Cre} x Ai9 mice. Right: confocal images showing AAV-SOM-EGFP expression (green) and SOM-tdTomato (red) in MEC deep layers of SOM^{Cre} x Ai9 mice. Scale bars, 50 μm.

b, Proportion of co-labeled cells in $EGFP^{+}$ cells (*n* = 6 slices from 3 mice). Error bars represent SEM.

c, Schematic illustrating virus strategy to express ChR2-mCherry in vCE L5a PCs and EGFP in vCE L5 SOM INs.

d, Left: widefield image showing the whole-cell recording of ChR2-positive neurons in vCE L5a. Scale bars, 50 μm. Right: representative traces displaying firing pattern (inset) and light-evoked action potentials (10 Hz) recorded from a ChR2-positive vCE L5a neuron. Scale bars (inset), 50 mV, 100 ms.

e, Representative traces displaying responses of L5a or L5b EGFP-positive neurons to light activation of L5a ChR2-positive neurons.

f, Summary plot exhibiting EPSC amplitude recorded from L5a (*n* = 8 cells from 5 mice) or L5b (*n* = 9 cells from 5 mice) EGFP-positive neurons. Error bars represent SEM.

g, Proportion of EGFP-positive cells responding to optical activation of ChR2-positive L5a PCs.

h, Proportion of MEC L5a (ChR2-negative and EGFP-negative), L2 or CA1 neurons responding to optical activation of ChR2-positive L5a PCs.

i, Summary plot exhibiting EPSC latencies vs. IPSC latencies recorded in MEC L5a PCs (ChR2-negative) or L2 neurons following activation of L5a ChR2-positive PCs (left, *n* = 7 cells from 4 mice; right, *n* = 5 cells from 4 mice).

Source data are provided as a Source Data file.

Supplementary Figure 8

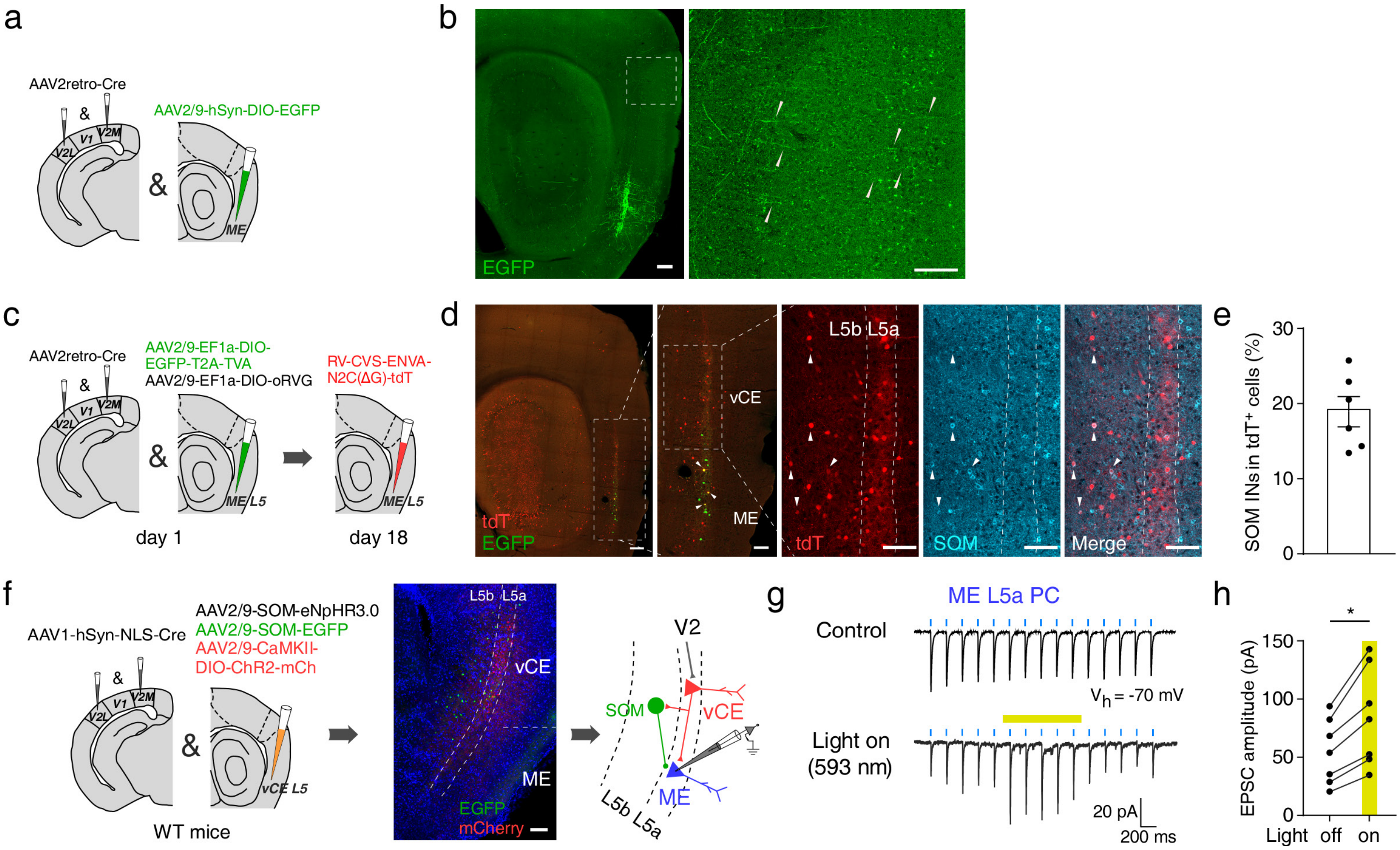

Supplementary Figure 8, related to Figure 6. L5b SOM INs are engaged in feedforward inhibition from vCE L5a to ME L5a.

a, Schematic illustrating viral injection strategy to label ME L5a neurons.

b, Confocal images showing the labeled ME L5a neurons and their axons in the dorsal MEC. Partial axons in the dorsal MEC were indicated by arrowheads. Scale bar, 200 μm (left), 100 μm (right).

c, Schematic illustrating retrograde trans-monosynaptic RV tracing from ME L5a.

d, Confocal images showing the expression of helper virus and RV within the MEC. Note starter cells (yellow, arrowheads) co-expressing TVA (green) and RV-CVS-ENVA-N2C(ΔG)-tdTomato (red) in ME L5a and their presynaptic neurons in vCE L5b (red) co-labeling with SOM antibody (cyan). Scale bars, 200 μm (left), 100 μm (others).

e, Proportion of SOM INs in vCE L5b RV-tdTomato-positive cells (n = 6 slices from 3 mice). Error bars represent SEM

f, Schematic illustrating virus strategy to express ChR2-mCherry in vCE L5a PCs and eNpHR3.0-EGFP in vCE L5 SOM INs for investigating feedforward inhibition in the DV pathway and confocal image showing SOM-EGFP and ChR2-mCherry expression in vCE L5. Scale bar, 100 μm.

g, Representative traces exhibiting effects of inhibiting SOM INs with yellow light (1 s) on blue light-evoked EPSCs (5 Hz) of a ME L5a PC.

h, Comparison of EPSC amplitudes of ME L5a PCs with yellow light off and on (n = 7 cells from 3 mice). *P = 0.0156, two-sided Wilcoxon signed-rank test.

Source data are provided as a Source Data file.

Supplementary Figure 9

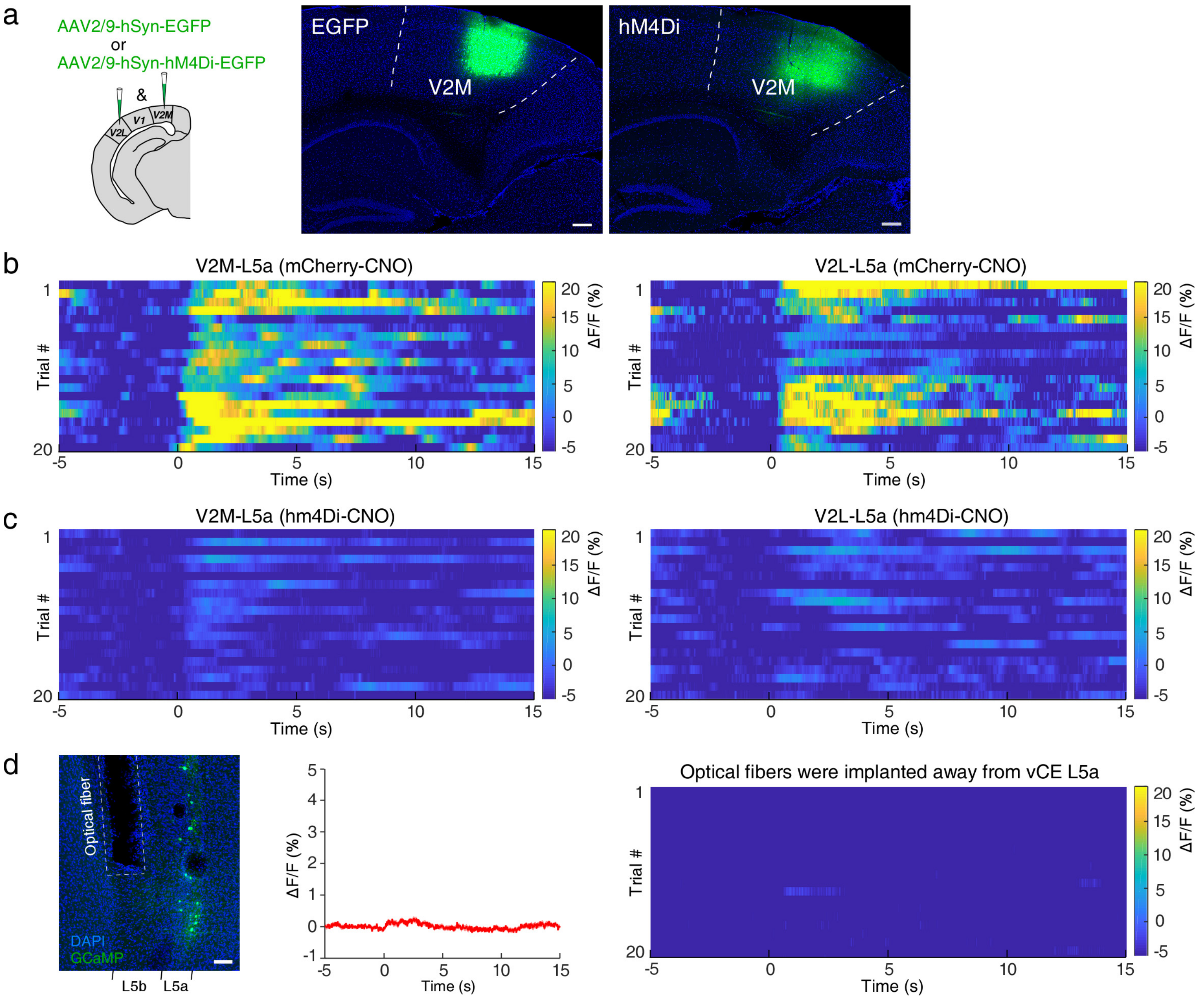

Supplementary Figure 9, related to Figure 7. The V2→MEC L5a pathway is critical for light stimulus-evoked responses in MEC L5a neurons.

a, Left: schematic illustrating the viral injection of EGFP or hM4Di-EGFP within V2M and V2L. Right: confocal images showing viral expression (green) within V2M. Scale bars, 500 μm.

b, Heatmaps showing the Ca^{2+} signals of vCE L5a neurons receiving V2M or V2L projections evoked by light stimuli from a CNO-treated mouse whose bilateral V2M or V2L neurons were infected with mCherry.

c, Heatmaps showing the Ca^{2+} signals of vCE L5a neurons receiving V2M or V2L projections evoked by light stimuli from a CNO-treated mouse whose bilateral V2M or V2L neurons were infected with hM4Di.

d, Left: representative image showing optical fiber placed away from vCE L5a. Scale bars, 100 μm. Middle: the averaged trace of Ca^{2+} recordings did not show significant light stimulus-evoked signals (n = 3 mice; 1 mouse from CNO-treated mCherry group and 2 mice from saline-treated hM4Di group). Right: Heatmap showing the Ca^{2+} recordings from a mouse with optical fiber placed away from vCE L5a.

Supplementary Figure 10

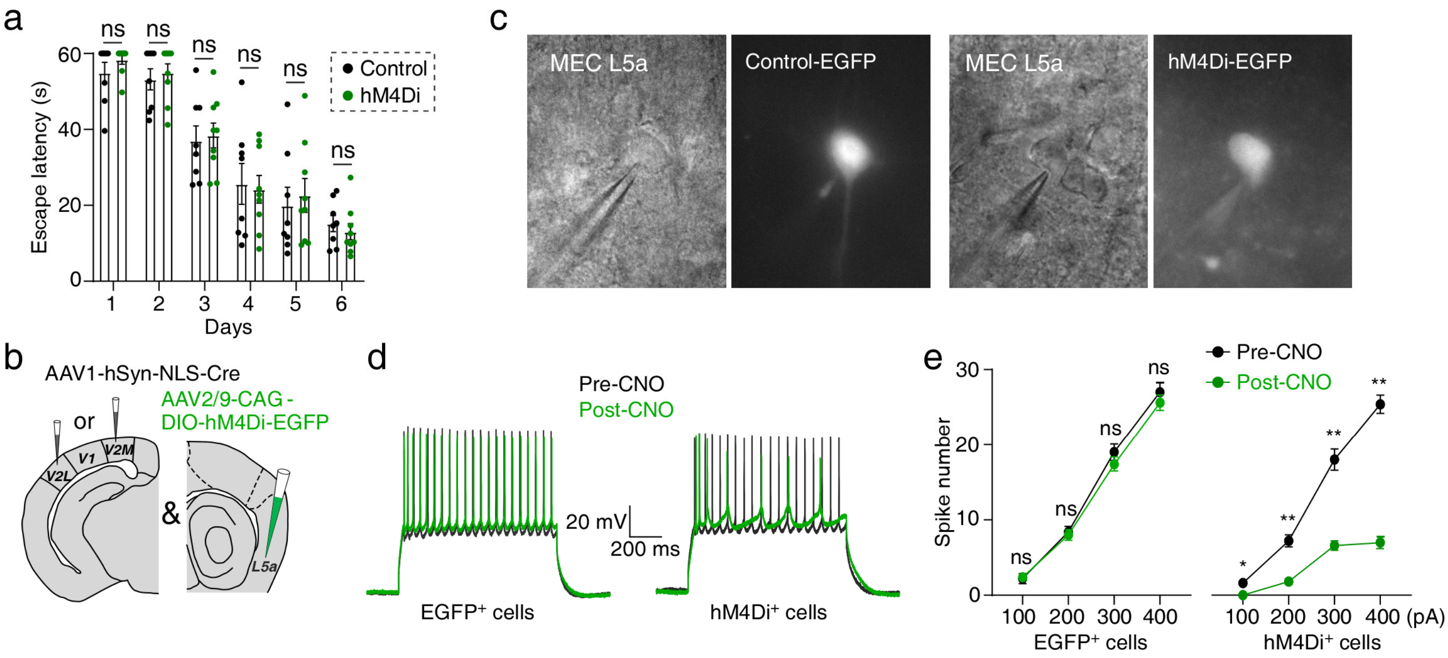

Supplementary Figure 10, related to Figure 7. The performance of the mice in the Morris water maze (MWM) training and *in vitro* validation of chemogenetics.

a, Escape latencies of the control group and the hM4Di group in the MWM test over six consecutive training days. The two groups showed no difference in learning to find the target platform. ns $P \geq 0.05$.

b, Schematic illustrating virus strategy to express EGFP or hM4Di-EGFP in vCE L5a neurons.

c, Widefield images under transmitted light (left) and fluorescence (right) microscopy showing whole-cell recordings of an EGFP-positive neuron (left) or an hM4Di-EGFP-positive neuron (right) in vCE L5a.

d, Representative traces demonstrating *in vitro* validation of DREADD-mediated inactivation on L5a neurons. CNO (5 µM) application suppressed the action potential firing induced by intrasomatic current injections in an hM4Di-EGFP-positive neuron but not in an EGFP-positive neuron.

e, Summary plot exhibiting the effect of CNO application on the action potential firing of EGFP-positive or hM4Di-EGFP-positive MEC L5a neurons (n = 5 cells from 3 mice/group). *P = 0.0476, **P = 0.0079, ns $P \geq 0.05$, two-sided Mann-Whitney U test. Error bars represent SEM. Source data are provided as a Source Data file.

Supplementary Figure 11

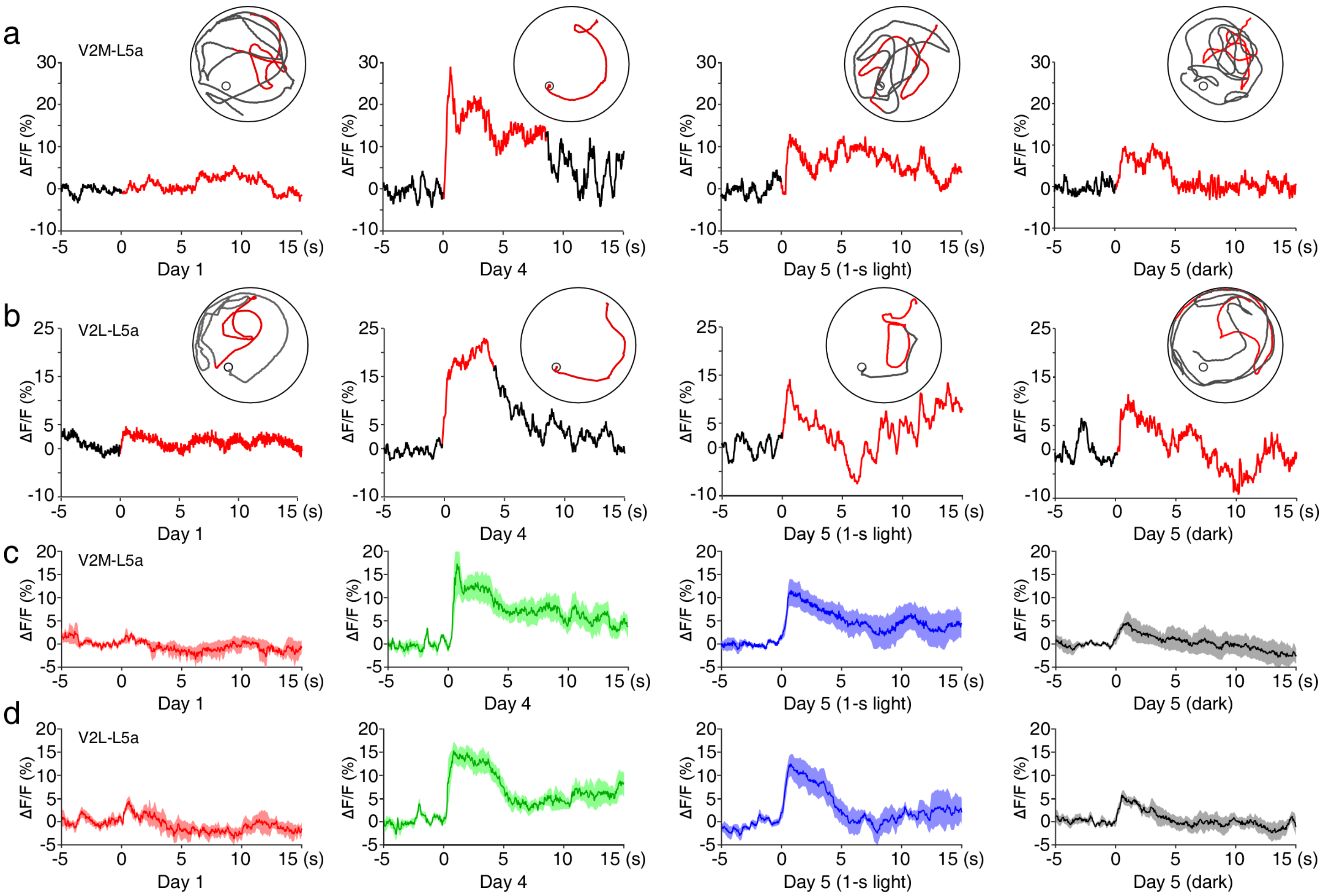

Supplementary Figure 11, related to Figure 7. Neural activity of MEC L5a neurons in the MWM tests.
a-b, Examples of the swim path (top) and the corresponding Ca^{2+} signals (bottom) in the vCE L5a neurons receiving V2M (**a**) or V2L (**b**) projections on day 1, day 4, and day 5 with 1-s light or in dark. The Ca^{2+} signal traces and their corresponding swim paths are marked in red.
c-d, Mean and SEM of Ca^{2+} signals in the vCE L5a neurons receiving V2M (**c**, $n = 5$ mice) or V2L (**d**, $n = 5$ mice) projections.

Supplementary Figure 12

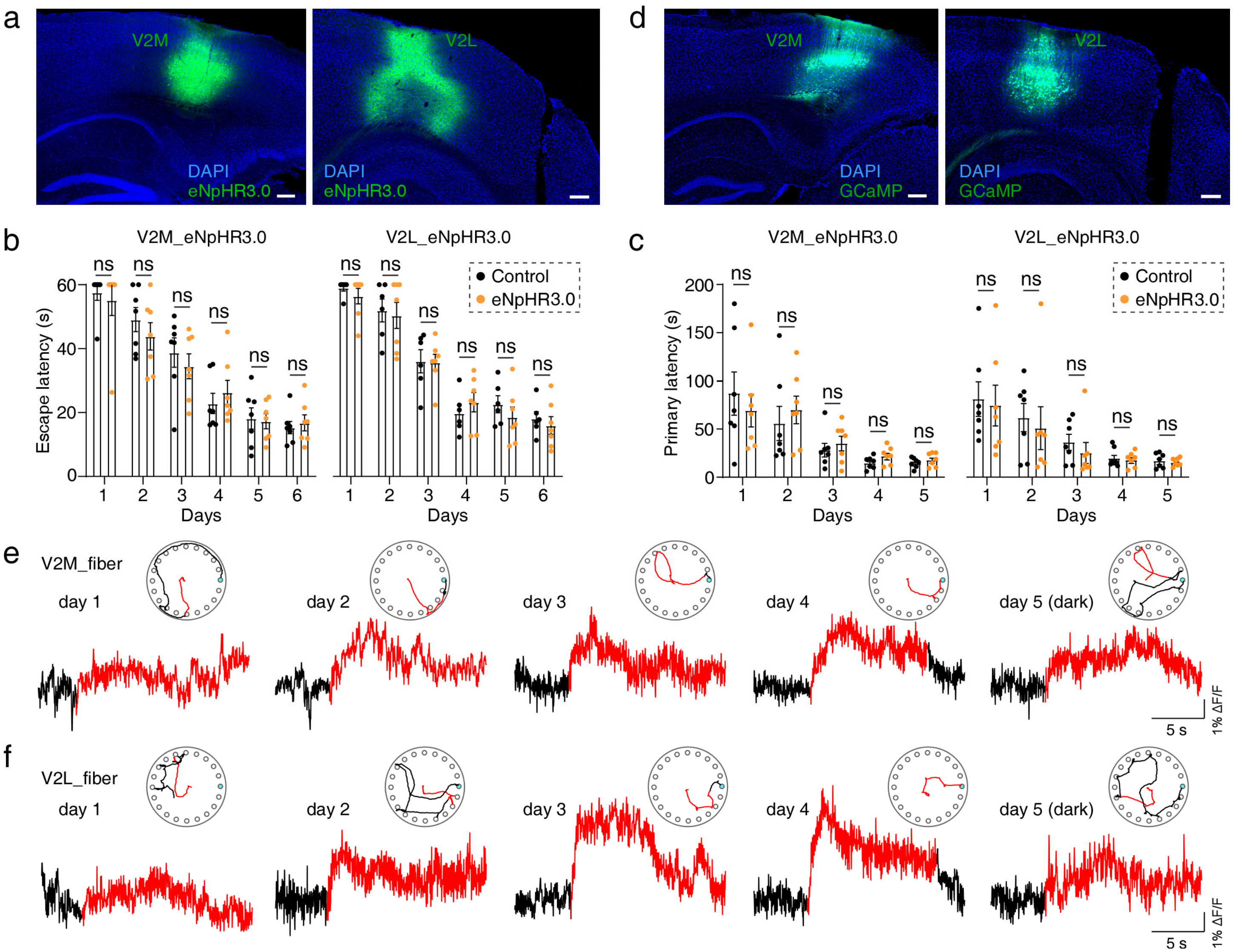

Supplementary Figure 12, related to Figure 8. The performance of the mice in the MWM and the Barnes maze (BM) training and the activity of V2 projections in MEC during the BM test.

a, Confocal images showing eNpHR3.0 viral injection site within V2M (left) and V2L (right). Scale bars, 200 μm.

b, Escape latencies of the control group and the eNpHR3.0 group in the MWM test over six consecutive training days, ns $P \geq 0.05$.

c, Primary latencies of the control group and the eNpHR3.0 group in the BM test over five consecutive training days, ns $P \geq 0.05$.

d, Confocal images showing GCaMP viral injection site within V2M (left) and V2L (right). Scale bars, 200 μm.

e-f, Examples of the movement trajectory (top) and the corresponding Ca^{2+} signals (bottom) of V2M fibers (**e**) or V2L fibers (**f**) on training days 1-4 and in the dark condition on day 5. The Ca^{2+} signal traces and their corresponding escape paths are marked in red.

Two-sided Mann-Whitney U test. Error bars represent SEM. Source data are provided as a Source Data file.
