# Supplementary material for: A non-canonical visual cortical-entorhinal pathway contributes to spatial navigation
Source: Nat Commun. 2024 May 15;15:4122. doi: 10.1038/s41467-024-48483-y (PMC11096324; doi:10.1038/s41467-024-48483-y)
Supplement: Supplementary file 3 — Reporting Summary [file 41467_2024_48483_MOESM3_ESM.pdf]

nature portfolio

Corresponding author(s): Guangfu Wang

Last updated by author(s): Mar 20, 2024

Reporting Summary

Nature Portfolio wishes to improve the reproducibility of the work that we publish. This form provides structure for consistency and transparency in reporting. For further information on Nature Portfolio policies, see our Editorial Policies and the Editorial Policy Checklist.

Statistics

For all statistical analyses, confirm that the following items are present in the figure legend, table legend, main text, or Methods section.

n/a	Confirmed	
☐	☒	The exact sample size (*n*) for each experimental group/condition, given as a discrete number and unit of measurement
☐	☒	A statement on whether measurements were taken from distinct samples or whether the same sample was measured repeatedly
☐	☒	The statistical test(s) used AND whether they are one- or two-sided<br>*Only common tests should be described solely by name; describe more complex techniques in the Methods section.*
☒	☐	A description of all covariates tested
☒	☐	A description of any assumptions or corrections, such as tests of normality and adjustment for multiple comparisons
☐	☒	A full description of the statistical parameters including central tendency (e.g. means) or other basic estimates (e.g. regression coefficient) AND variation (e.g. standard deviation) or associated estimates of uncertainty (e.g. confidence intervals)
☐	☒	For null hypothesis testing, the test statistic (e.g. *F*, *t*, *r*) with confidence intervals, effect sizes, degrees of freedom and *P* value noted<br>*Give P values as exact values whenever suitable.*
☒	☐	For Bayesian analysis, information on the choice of priors and Markov chain Monte Carlo settings
☒	☐	For hierarchical and complex designs, identification of the appropriate level for tests and full reporting of outcomes
☒	☐	Estimates of effect sizes (e.g. Cohen's *d*, Pearson's *r*), indicating how they were calculated

Our web collection on statistics for biologists contains articles on many of the points above.

Software and code

Policy information about availability of computer code

Data collection	Confocal images: ZEN Black 3.0 (Zeiss); electrophysiological data: Igor Pro 6.0 (WaveMetrics); calcium signal data: Tripple Color Multi-Channel Fiberphotometry 2.0 (Thinker Tech); behavioral data: Smart 3.0 (Panlab Harvard Apparatus).
Data analysis	ZEN 2.6 (Zeiss); ImageJ 1.53 (National Institutes of Health); Igor Pro 6.0 (WaveMetrics); Neurolucida 11 (MBF Bioscience); Tripple Color Multi-Channel Analysis Package 4.0 (Thinker Tech); Smart 3.0 (Panlab Harvard Apparatus); GNU Octave 7.1.0 (John W. Eaton); Python 3.8 (Python Software Foundation); RStudio 1.4.1717 (Posit); Microsoft Excel 2019 (Microsoft).

For manuscripts utilizing custom algorithms or software that are central to the research but not yet described in published literature, software must be made available to editors and reviewers. We strongly encourage code deposition in a community repository (e.g. GitHub). See the Nature Portfolio guidelines for submitting code & software for further information.

Data

Policy information about availability of data

All manuscripts must include a data availability statement. This statement should provide the following information, where applicable:

- Accession codes, unique identifiers, or web links for publicly available datasets
- A description of any restrictions on data availability
- For clinical datasets or third party data, please ensure that the statement adheres to our policy

All data supporting the results of this study are available within the paper and its Supplementary Information. Raw data are too large to be publicly shared but are available from the corresponding author upon reasonable request. Source data are provided with this paper.

Research involving human participants, their data, or biological material

Policy information about studies with human participants or human data. See also policy information about sex, gender (identity/presentation), and sexual orientation and race, ethnicity and racism.

Reporting on sex and gender	N/A.
Reporting on race, ethnicity, or other socially relevant groupings	N/A.
Population characteristics	N/A.
Recruitment	N/A.
Ethics oversight	N/A.

Note that full information on the approval of the study protocol must also be provided in the manuscript.

Field-specific reporting

Please select the one below that is the best fit for your research. If you are not sure, read the appropriate sections before making your selection.

☒ Life sciences ☐ Behavioural & social sciences ☐ Ecological, evolutionary & environmental sciences

For a reference copy of the document with all sections, see nature.com/documents/nr-reporting-summary-flat.pdf

Life sciences study design

All studies must disclose on these points even when the disclosure is negative.

Sample size	The sample sizes were determined based on those reported in previous works of the field [Refs 21, 22, 23, 31, 32 and 55]. No statistical methods were used to predetermine sample sizes.
Data exclusions	No data was excluded from analyses except that the injections missed the target areas of brain. Paxinos and Franklin's the Mouse Brain in Stereotaxic Coordinates was used to identify brain areas.
Replication	All anatomical, physiological and behavioral experiments were successfully replicated from different animals and/or multiple tissue sections across different anatomical levels of cortical areas (>= 3 animals). Independent experiments were performed on different days.
Randomization	Mice were randomly selected from both sexes and randomly allocated to experimental groups for all the experiments.
Blinding	For viral tracing, data collection and analysis were performed by different experimenters. For morphological reconstructions of L5a pyramidal neurons, the recordings and the reconstructions were conducted by different experimenters, and the experimenters conducting morphological reconstructions were completely blinded to the purpose of the experiment. For in vitro electrophysiological recordings, blinding was not relevant because cell type was evident from the fluorescence and the location in brain slices. For in vivo experiments, the experimenter was blinded to the group allocation before data collection and analysis.

Reporting for specific materials, systems and methods

We require information from authors about some types of materials, experimental systems and methods used in many studies. Here, indicate whether each material, system or method listed is relevant to your study. If you are not sure if a list item applies to your research, read the appropriate section before selecting a response.

Materials & experimental systems

n/a	Involved in the study
☐	☒ Antibodies
☒	☐ Eukaryotic cell lines
☒	☐ Palaeontology and archaeology
☐	☒ Animals and other organisms
☒	☐ Clinical data
☒	☐ Dual use research of concern
☒	☐ Plants

Methods

n/a	Involved in the study
☒	☐ ChIP-seq
☒	☐ Flow cytometry
☒	☐ MRI-based neuroimaging

Antibodies

Antibodies used

Primary antibodies:
Mouse anti-c-Fos IgG (1:500; Abcam, ab208942)
Rat anti-Ctip2 IgG (1:500; Abcam, ab18465)
Rabbit anti-GAD65/67 IgG (1:500; Abcam, ab11070)
Rabbit anti-CaMKII IgG (1:500; Abcam, ab52476)
Rabbit anti-PCP4 IgG (1:500; Sigma, HPA005792)
Rat anti-Somatostatin IgG (1:200; Millipore, MAB354)

Secondary antibodies:
Alexa-488-conjugated goat anti-mouse IgG (1:1000; Abcam, ab150113)
Alexa-488-conjugated donkey anti-rabbit IgG (1:1000; Abcam, ab150073)
Alexa-405-conjugated goat anti-rabbit IgG (1:1000; Abcam, ab175652)
Alexa-555-conjugated goat anti-rabbit IgG (1:1000; Abcam, ab150078)
Alexa-555-conjugated goat anti-mouse IgG (1:1000; Cell signaling technology, #4409)
Alexa-647-conjugated goat anti-mouse IgG (1:1000; Cell signaling technology, #4410)
Alexa-405-conjugated donkey anti-rat IgG (1:1000; Abcam, ab175670)

Validation

These antibodies have been validated by the manufacturers and numerous scientists. The results and representative citations are available in the websites of the manufactures as follows:
Mouse anti-c-Fos IgG (Abcam, ab208942): https://www.abcam.com/products/primary-antibodies/c-fos-antibody-2h2-ab208942.html
Rat anti-Ctip2 IgG (Abcam, ab18465): https://www.abcam.com/products/primary-antibodies/ctip2-antibody-25b6-ab18465.html
Rabbit anti-GAD65/67 IgG (Abcam, ab11070): https://www.abcam.com/products/primary-antibodies/gad65--gad67-antibody-ab11070.html
Rabbit anti-CaMKII IgG (Abcam, ab52476): https://www.abcam.com/products/primary-antibodies/camkii-antibody-ep1829y-ab52476.html
Rabbit anti-PCP4 IgG (Sigma, HPA005792): https://www.sigmaaldrich.com/US/en/product/sigma/hpa005792
Rat anti-Somatostatin IgG (Millipore, MAB354): https://www.sigmaaldrich.com/US/en/product/mm/mab354

Animals and other research organisms

Policy information about studies involving animals; ARRIVE guidelines recommended for reporting animal research, and Sex and Gender in Research

Laboratory animals

Adult male and female mice (2–4 months) were used for all experiments. The Cre and Ai9 reporter lines were purchased from Jackson Laboratory (Vgat-Cre, #028862; SOM-Cre, #013044; PV-Cre, #017320; Ai9, #007909). The Vgat-Cre x Ai9, SOM-Cre x Ai9 and PV-Cre x Ai9 mice were generated by crossing the corresponding Cre line with the Ai9 line.

Wild animals

No wild animals were used.

Reporting on sex

Female and male mice were used. No sexual difference was hypothesized in this study and sex was not considered in the study design. This information has not been constantly collected.

Field-collected samples

No field collected samples were used.

Ethics oversight

All procedures for animal surgery and maintenance were performed following protocols approved by the Institutional Animal Care and Use Committee (IACUC) of Harbin Institute of Technology.

Note that full information on the approval of the study protocol must also be provided in the manuscript.

Plants

Seed stocks	N/A.
Novel plant genotypes	N/A.
Authentication	N/A.
